# Gut Microbiota as a Potential Player in Mn-Induced Neurotoxicity

**DOI:** 10.3390/biom11091292

**Published:** 2021-08-31

**Authors:** Alexey A. Tinkov, Airton C. Martins, Daiana Silva Avila, Victor A. Gritsenko, Anatoly V. Skalny, Abel Santamaria, Eunsook Lee, Aaron B. Bowman, Michael Aschner

**Affiliations:** 1 Laboratory of Ecobiomonitoring and Quality Control, Yaroslavl State University, 150003 Yaroslavl, Russia; tinkov.a.a@gmail.com; 2 Laboratory of Molecular Dietetics, IM Sechenov First Moscow State Medical University (Sechenov University), 119435 Moscow, Russia; skalnylab@gmail.com; 3 Department of Molecular Pharmacology, Albert Einstein College of Medicine, Bronx, NY 10461, USA; airton.dacunhamartinsjunior@einsteinmed.org; 4 Laboratory of Biochemistry and Toxicoology in Caenorhabditis elegans, Universidade Federal do Pampa, Campus Uruguaiana, BR-472 Km 592, Uruguaiana 97500-970, RS, Brazil; avilads1@gmail.com; 5 Institute of Cellular and Intracellular Symbiosis, Ural Branch of the Russian Academy of Sciences, Pionerskaya st 11, 460000 Orenburg, Russia; vag59@mail.ru; 6 Laboratory of Medical Elementology, KG Razumovsky Moscow State University of Technologies and Management, 109004 Moscow, Russia; 7 Laboratorio de Aminoácidos Excitadores, Instituto Nacional de Neurología y Neurocirugía, Mexico City 14269, Mexico; absada@yahoo.com; 8 College of Pharmacy and Pharmaceutical Sciences, Florida A&M University, Tallahassee, FL 32307, USA; eunsook.lee@famu.edu; 9 School of Health Sciences, Purdue University, West Lafayette, IN 47907, USA; bowma117@purdue.edu

**Keywords:** manganese, gut microbiome, bacterial metabolites, lipopolysaccharide, neurotoxicity

## Abstract

Manganese (Mn) is an essential metal, which at high exposures causes neurotoxic effects and neurodegeneration. The neurotoxic effects of Mn are mediated by neuroinflammation, oxidative and endoplasmic reticulum stress, mitochondrial dysfunction, and other mechanisms. Recent findings have demonstrated the potential impact of Mn overexposure on gut microbiota dysbiosis, which is known to contribute to neurodegeneration via secretion of neuroactive and proinflammatory metabolites. Therefore, in this review, we discuss the existing data on the impact of Mn exposure on gut microbiota biodiversity, bacterial metabolite production, and gut wall permeability regulating systemic levels. Recent data have demonstrated that Mn exposure may affect gut microbiota biodiversity by altering the abundance of Shiegella, Ruminococcus, Dorea, Fusicatenibacter, Roseburia, Parabacteroides, Bacteroidetes, Firmicutes, Ruminococcaceae, Streptococcaceae, and other bacterial phyla. A Mn-induced increase in Bacteroidetes abundance and a reduced Firmicutes/Bacteroidetes ratio may increase lipopolysaccharide levels. Moreover, in addition to increased systemic lipopolysaccharide (LPS) levels, Mn is capable of potentiating LPS neurotoxicity. Due to the high metabolic activity of intestinal microflora, Mn-induced perturbations in gut microbiota result in a significant alteration in the gut metabolome that has the potential to at least partially mediate the biological effects of Mn overexposure. At the same time, a recent study demonstrated that healthy microbiome transplantation alleviates Mn-induced neurotoxicity, which is indicative of the significant role of gut microflora in the cascade of Mn-mediated neurotoxicity. High doses of Mn may cause enterocyte toxicity and affect gut wall integrity through disruption of tight junctions. The resulting increase in gut wall permeability further promotes increased translocation of LPS and neuroactive bacterial metabolites to the systemic blood flow, ultimately gaining access to the brain and leading to neuroinflammation and neurotransmitter imbalance. Therefore, the existing data lead us to hypothesize that gut microbiota should be considered as a potential target of Mn toxicity, although more detailed studies are required to characterize the interplay between Mn exposure and the gut, as well as its role in the pathogenesis of neurodegeneration and other diseases.

## 1. Introduction

Neurotoxicity is a process where chemical, biological, or physical agents trigger biochemical changes in nervous system cells, leading to an impairment in neuronal functions. Damage causing atrophy, progressive loss of function, and death of neurons or glial cells is referred to as neurodegeneration. Common neurotoxins include pesticides, solvents, drugs of abuse, and metals such as manganese (Mn) [1].

Mn neurotoxicity has been known of for over 100 years, and it has been predominantly observed in occupational settings most often by inhalation or non-occupationally by ingestion of high levels [2]. The dopaminergic areas of the brain are particularly susceptible to an excess of this metal. The first reports of Mn neurotoxicity, or *locura manganica,* were described following occupational exposure to black Mn oxide grinding, characterized in workers by the development of postural and gait alterations and muscular weakness. Mn neurotoxicity has most often been associated with occupations such as Mn mining and smelting, as well as battery and steel production [3,4]. Non-occupational exposures occur mainly from contaminated drinking water, milk and infant formulas, total parenteral nutrition (TPN), the air as a result of the combustion of the gasoline additive methylcyclopentadienyl manganese tricarbonyl (MMT), and the use of drugs that contain Mn as methcathinone [3,5,6,7]. The US Food and Drug Administration (FDA) recommends a daily value for Mn of 2.3 mg for adults [8]. Exceeding Mn levels in drinking water and dietary products, especially in the highly bioavailable form, may result in adverse health effects. Thus, regulation of dietary Mn intake should be monitored to avoid toxic effects [9].

Many studies indicate that manganism symptoms are caused by the accumulation of Mn in brain areas that are rich in dopaminergic (DAergic) neurons, which are largely involved in motor control, notably the striatum. Upon Mn intoxication, there is a primary increase in DA production, which has been associated with psychiatric symptoms that have influenced the denomination of the condition known as *locura manganica* [4,6]. As Mn poisoning progresses, catecholamine levels decrease due to the loss of DAergic neurons, resulting in a parkinsonian-like syndrome [10]. The similarities between the clinical manifestations of Parkinson’s disease (PD) and manganism include the presence of generalized bradykinesia and body rigidity caused by the loss of DAergic neurons in the basal ganglia. Differences include the distinct basal ganglia areas affected; the substantia nigra of the pars compacta is spared in manganism. Therefore, there are less frequent resting tremors, but a particular propensity to falling backwards. However, whether manganism patients are irresponsive to levodopa has yet to be fully determined [6].

Most recently, a microbiological factor has been associated with neurodegenerative processes: gut microbiota dysbiosis, where the balance between beneficial and pathogenic bacteria is disrupted. Notably, human gut microbiota is a complex assembly of microorganisms that can produce molecules that interfere with host physiology [11]. A healthy commensal relationship affords benefits to the associated organisms. However, dysbiosis can impair the production of important molecules, such as short-chain fatty acids, and allow the growth of pathogenic bacteria [12]. It has been demonstrated that alterations in enteric bacterial components and/or metabolites that reach the central nervous system (CNS) may elicit neuroinflammation and/or neurodegeneration [13,14,15,16]. For instance, it has been demonstrated that Alzheimer’s disease and Parkinson’s disease patients display dysbiosis, which is likely involved in these diseases’ pathogenesis through mechanisms that include blood–brain barrier destruction, inflammation, and oxidative stress, affecting behavior and cognitive functions [15,17]. Of note, the Braak hypothesis was the first association between the gut and Parkinson’s disease, suggesting that this disease initiates in the gut before affecting the brain, when the symptoms become noticeable [18]. Nonetheless, the Braak hypothesis requires further validation, particularly in the cause–effect relationships of gut to brain progression and etiology. Evidence also suggests that gut colonization of pathogenic bacteria can increase polyglutamine proteotoxicity [16].

Alterations in gut microbiota can be triggered by a wide range of drugs and environmental factors. It has been demonstrated that Mn, besides altering the DAergic system, can alter gut permeability and gut microbiota, therefore causing dysbiosis and leading to the release of neurotoxic metabolites, which could contribute to parkinsonism-like and psychological symptoms caused by Mn exposure. In this review we will discuss the relationship between Mn exposure, the gut microbiome, and neurotoxicity.

## 2. A Brief Introduction to Mn Neurotoxicity: Gut-Independent Mechanisms

Several mechanisms by which Mn causes neurotoxicity have been unraveled, but it is still considered poorly understood. Multiple analyses of pathways affected by Mn exposure using metallomics, proteomics, gene expression, and bioinformatics revealed that Mn exposure alters several processes such as proteostasis, cell metabolism and signaling, immunity and inflammation, cell cycle, and neurodegeneration-associated pathways [19]. It is well established that Mn accumulates in areas rich in dopaminergic neurons. In these areas, Mn reacts with DA through the Fenton reaction, causing DA oxidation, which triggers the formation of reactive oxygen species (ROS) and DA-o-quinone, thus leading to oxidative damage and DAergic neuronal death [10,20]. Various studies point out that once inside DAergic neurons, the mitochondria are the main cellular targets of Mn toxicity. This selective uptake by DAergic neurons and sequestration by mitochondria are attributed to transport mechanisms that favor the influx of Mn over its efflux, causing the Mn accumulation in this organelle and therefore altering its function [21]. This has been confirmed by imaging techniques. Mn accumulation in the mitochondria of neurons and astrocytes precedes any structural damage to these cells [22]. Once inside, Mn can impair the electron transfer chain by inhibiting complexes I-IV of the electron transporter chain and interfering with oxidative phosphorylation [23,24,25]. Mn can also bind to a specific Mg^2+^ site on the F1-ATPase, slowing its activity. Moreover, Mn can alter calcium homeostasis in mitochondria by inhibiting its efflux and increasing its levels in this organelle [21,26]. Altogether, these effects lead to reduced energy production and increased ROS levels (notably O^2−^) by deregulating the electron transport chain, elevating intracellular calcium levels, and modifying the inner mitochondrial membrane potential. This leads to the opening of the mitochondrial permeability transition pore, with ensuing mitochondrial dysfunction [27].

Additionally, calcium-induced mitochondrial permeability transition (MPT) opening leads to the activation of the B-cell lymphoma 2 (Bcl-2) family of proteins, especially Bax/Bac. When these proteins are activated, they induce the release of cytochrome c (Cyt c) from the mitochondria [28]. Cyt c activates, via the ERK pathway, the cysteine protease caspase-3, a pro-apoptotic protein that causes chromatin condensation and DNA fragmentation. Indeed, Mn exposure in astrocytes results in ERK and caspase-3 activation [29], and DNA strand breakage in striatal neurons was observed at low Mn exposure [27].

Mn can also promote autophagy dysfunction by altering Atg5 and Beclin-1 protein levels in vivo and in vitro. Studies have demonstrated a paradoxical effect caused by this metal, since either an increase or decrease in this process can be promoted by Mn depending on the time of exposure [30,31]. Several studies point to the upregulation of autophagy proteins by Mn, suggesting either increased autophagic flux or blocked autophagic clearance. Notably, both overactivation and suppression of autophagy will induce an autophagy-dependent programmed cell death [32]. Autophagy is a major process, since cells can degrade misfolded proteins and damaged organelles, reorganizing cellular structure and function [33,34,35]. It has been demonstrated that Mn can increase the affinity between Bcl-2-Beclin-1, impairing the autophagic process in longer exposures. Mn can induce nitrosative stress, which can further inhibit Bcl-2 phosphorylation and cause Beclin-1 release [36].

Mn also induces endoplasmic reticulum stress, which can lead to apoptosis and autophagy deregulation [37,38]; neuroinflammation, astrocytes being the main mediators of the proinflammatory effects of the metal [19,39]; alterations in cell signaling such as AKT, JNK, and other insulin-like pathways (this last one in *Caenorhabditis elegans*) [19,40,41]; and epigenetic alterations by increasing DNA methylation and histone acetylation in different models in vitro and in vivo [40,41,42]. Mn also interferes with GABAergic and catecholaminergic neurotransmissions by reducing the production of the neurotransmitters. Glutamate signaling is remarkably altered by Mn, since it can decrease the expression of GLAST and GLT-1, therefore reducing glutamate uptake [43]. It also inhibits glutamine synthetase and up-regulates phosphate-activated glutaminase, resulting in a significant increase of glutamate and a decline in glutamine levels in the hippocampus, thalamus, striatum, and globus pallidus of Mn-exposed rats [44].

This section briefly demonstrated the wide plethora of molecular and cellular alterations that Mn exposure can cause through neurotoxicity and neurodegeneration. Recently, additional evidence has been pointing to other mechanisms related to gut–brain crosstalk that may help to explain several physiological hallmarks of Mn-induced neurotoxicity, which we will discuss in the next sections.

## 3. The Role of Gut Microbiota and Its Metabolites in Brain Functioning and Neurodegeneration

The microbiome consists of the collective genomes of all the microorganisms, such as bacteria, archaea, viruses, and fungi, that are present in the human body. The gut microbiota represent trillions of microorganisms, symbiotic and pathogenic, that colonize the human gastrointestinal tract [45]. In addition, metabolites, which are products of microbial metabolism, may be important to the development and function of the immune, endocrine, and neurological systems [46]. Growing evidence suggests that gut microbiota play an important role in neurochemical pathways through the microbiota–gut–brain axis. They modulate stress responses and neurotransmitter production in the central nervous system (CNS), such as catecholamines, serotonin, and glutamate. They also affect neurogenesis in various regions of the brain, as well as neurodevelopment and health [47,48]. Furthermore, alterations in the composition, variety, and abundance of microorganisms and metabolites in the gastrointestinal tract may lead to the onset of neurodegenerative disease [13,49,50].

Several neurodegenerative diseases, such as Alzheimer’s disease (AD), have been posited to be related to gut microbiota composition. For example, it has been suggested that gut microbiota may modulate amyloid-β accumulation, alter blood–brain barrier permeability, and induce neuroinflammation via its metabolites [51,52]. In fact, several microbiota species, such as enterobacteria, can secrete amyloid protein curli, which accelerate amyloid polymerization and create potent immunogenic complexes that activate immune cells [49]. Moreover, Gram-negative bacteria are the primary producers of Aβ prion-like proteins and lipopolysaccharides (LPS), which are elevated in the brains of AD patients [50]. Indeed, the microbiota–gut system is a site of Aβ production and LPS formation, where enterocytes are able to accumulate substantial amounts of Aβ and LPS, which are integrated into ApoE proteins and, through the blood stream, enter the brain [53,54,55].

Clinical studies have demonstrated an association between the phenotype of PD and gut microbial dysbiosis [56,57,58]. Indeed, it has been demonstrated that PD patients have a significant decrease of *Prevotellaceae* in their feces as compared to control individuals. Additionally, postural instability and gait difficulty were associated with an abundance of PD patients, suggesting that the intestinal microbiome is changed in PD patients and is associated with the motor phenotype [56]. Likewise, a recent cohort study reported a significant increase in three bacterial families in PD patients compared to controls [59]. Furthermore, the number of bacteria that facilitate fermentation of cellulose in the colon and produce short chain fatty acids (SCFAs) had decreased, while putative pathobionts that produce endotoxins and promote inflammation in human intestines had increased in the PD group compared to control individuals. The authors suggested that structural changes to gut microbiota with reduced production of SCFAs and enhanced production of endotoxins and neurotoxins may be associated with the development of PD pathology [60]. Corroborating the clinical studies, Sun et al., (2018), using sequencing of 16S rRNA, identified differences in gut microbiota between PD mice and normal mice. It was reported that phylum *Firmicutes* and order *Clostridiales* decreased in PD mice feces while phylum *Proteobacteria*, which is related to gut inflammation, was enhanced in PD mice. According to the authors, gut microbial dysbiosis may be involved in PD pathogenesis and clinical manifestations [61].

Gut microbial dysbiosis can be affected by multiple environmental pollutants such as microplastics, pesticides, persistent organic pollutants, nanoparticles, metals, and metalloids [62,63,64,65,66].

## 4. Mn and Gut Microbiota

Mn was shown to interfere with gut functions through dysregulation of gut wall integrity and gut microbiota biodiversity, as well as metabolic function of the latter. However, despite the well-known essentiality of Mn for bacteria, the impact of Mn exposure on gut microflora has been studied only recently.

Mn can affect gut microbiota biodiversity in mice, revealing its sex-specific effects. Particularly, female mice responded to oral Mn exposure (20 mg/kg body weight/day with drinking water for 13 weeks) with a significant decrease in Firmicutes and a tendency toward an elevated number of Bacteroidetes. Inversely, in male C57BL/6 mice, a Mn overload was associated with a significant decrease in the relative abundance of Bacteroidetes, whereas the number of Firmicutes significantly increased. The observed changes in female mice were associated with a significant up-regulation of LPS biosynthesis genes. In addition, Mn exposure also significantly affected GABA and glutamate production by gut bacteria in fecal samples [67].

Although unrelated to ulcerative colitis or Crohn’s disease, dietary Mn levels with a mean of 6–7 mg/day were found to be inversely associated with Shiegella and Ruminococcus in Crohn’s disease intestinal biopsies, whereas in ulcerative colitis subjects, they were directly associated with the abundance of Dorea, Fusicatenibacter, and Roseburia (biopsy), as well as Parabacteroides (fecal samples) [68].

The modulatory effects of Mn on gut microbiomes were also demonstrated in non-mammalian species. Particularly, in breeding geese during their laying period, supranutritional doses of Mn (30 mg/kg Mn for 10 weeks) increased the abundance of Bacteroidetes, Bacteroidaceae, Bacteroides, and Ruminococcaceae, whereas the relative number of Streptococcaceae decreased [69]. It is also notable that Mn-induced alterations of gut microbiota were also observed in Lymantria dispar asiatica (Lepidoptera: Erebidae) larvae fed a 0.40 mmol MnCl_2_/g diet for 84 h [70].

Due to the high metabolic activity of intestinal microflora, Mn-induced perturbations in gut microbiota result in a significant alteration of gut metabolome. Specifically, preliminary data demonstrated the impact of oral Mn overload (15 mg/kg/day MnCl_2_ for 30 days via oral gavage) on fecal metabolomics, including reduced stool butyrate, α-tocopherol, and cholestane levels, as well as increased palmitic and cholic acid levels in rat feces that may, at least partially, mediate the effects of Mn in the organism [71]. Correspondingly, a recent study demonstrated that—in parallel with neurodegeneration, amyloid accumulation, neuronal apoptosis, and necrosis—oral exposure to 200 mg/L of Mn via drinking water for 5 weeks results in reduced gut biodiversity and alters tryptamine, taurodeoxycholic, β-hydroxypyruvic, and urocanic acid metabolism. At the same time, healthy microbiome transplantation alleviated Mn-induced neurotoxicity, being indicative of the significant role of gut microflora in the cascade of Mn neurotoxicity [72]. Moreover, studies have shown Mn-induced increases in Aβ and Tau production, inflammatory cytokines such as IL-1β, and NLRP3 inflammasome, leading to hippocampal neurodegeneration in rat brain. These effects were closely associated with increased inflammation in peripheral blood and gut dysbiosis. Fecal microbiome transplantation from normal rats attenuated Mn-induced neurotoxicity by downregulating Aβ and Tau expression and inhibiting cerebral NLRP3 inflammasome in rat brain [73]. Taken together with increased gut permeability upon Mn overexposure, gut dysbiosis and the resulting alteration of intestinal metabolome may result in the increased transfer of bacterial metabolites, including neuroactive metabolites, into the brain and other tissues.

The impact of Mn overexposure on gut microbiota results in increased LPS production, due to a higher number of Bacteroidetes, which are known as key contributors to circulating LPS levels [74]. The latter was shown to play a significant role in neurotoxicity and neurodegeneration due to its proinflammatory activity [75]. In addition, Mn was also shown to interfere with LPS neurotoxicity in primary neuronal and neuron-glia cultures exposed to 30 μM MnCl_2_ and 2 ng/mL LPS [76]. Therefore, it is proposed that Mn overexposure may not only increase LPS translocation due to dysbiosis and increased gut wall permeability but also potentiate LPS neurotoxicity and neuroinflammation.

Similarly to Mn, gut dysbiosis may significantly contribute to the neurotoxicity of other metals [66]. For instance, lead (Pb) and cadmium (Cd) exposure has been reported to change microbial biodiversity and cause dysbiosis of gut microbiota. Pyrosequencing of 16S RNA sequences showed changes in bacterial commensal communities in rats exposed to Pb and Cd over 8 weeks when compared to a control group [77]. Methylmercury (MeHg) induces alterations in intestinal microbial communities, as well as changes in intestinal neurotransmitters and metabolites, suggesting a potential association between gut microbiota and MeHg-induced neurotoxicity [78]. Moreover, the involvement of microbiota dysbiosis in neurotoxicity was evaluated at ecologically relevant Cd concentrations in zebrafish, showing significant changes in bacterial loads involved in the regulation of neurodegenerative diseases [79]. Although evidence shows association between heavy metal exposure and the alteration of gut microbiota, how gut microbial dysbiosis contributes to neurotoxicity and neurodegeneration is yet to be established. Further studies are required to understand the potential factors regulating the microbiota–gut–brain axis.

## 5. Mn and Gut Wall Permeability

Along with the secretion of bacterial neuroactive metabolites, due to the altered taxonomic characteristics of gut microbiota, systemic levels of these bioactive molecules are also regulated by gut permeability. Therefore, the impact of Mn on gut permeability and the responsible tight junction proteins is also discussed as the potential mediator of microbiome–brain interaction.

Corroborating the role of physiological doses of Mn in gut wall integrity, a recent study demonstrated that Mn deficiency (0, 5, and 15 ppm Mn in diet for 7 days) results in increased gut permeability due to tight junction protein dysfunction and aggravates dextran sulfate sodium-induced colitis in mice [80]. However, no significant alteration of gut microbiota was observed upon Mn deficiency [81].

A study in grass carp (*Ctenopharyngodon idellus*) demonstrated that both deficiency (3.65 mg/kg diet) and excess (≥18.24 mg/kg diet) of Mn result in intestinal inflammation, oxidative stress, and reduced tight junction proteins (claudin-b, claudin-c, claudin-15, occludin, and zonula occludens-1), whereas improvement of Mn levels reversed these effects [82]. Similar effects were observed in oriental river prawn *Macrobrachium nipponense* (De Haan), characterized by altered intestinal morphology upon both Mn deficiency (5.4 mg/kg diet) and excess (150 mg/kg diet) [83].

Correspondingly, missense mutation A391T of the SLC39A8 gene, which is known as a Mn transporter, was associated with profound Mn deficiency and altered the intestinal mucus barrier function in mice. The latter is expected to be related to altered glycoprotein structures, including glycocalyx, through the modulation of Mn-dependent glycosyltransferases [84]. In multivariate models, this mutation was also shown to be associated with a predominant increase in the abundance of Firmicutes [85].

It has also been demonstrated that Mn oxide nanoparticles with a diameter of 16.8 ± 2.6 nm were shown to potentiate E. coli LF82 lysate-induced damage to the intestinal epithelial cell model MODE-K through inhibition of reparative processes, induction of proinflammatory cytokine production, and mitochondrial dysfunction [86].

Therefore, the existing data demonstrate that Mn plays a significant role in the maintenance of gut wall integrity, whereas both Mn deficiency and excess result in impaired enterocyte tight junctions and increased gut permeability, due to the role of physiological Mn levels in cell metabolism and cytotoxicity of Mn overload, respectively.

## 6. Mn as a Player in Nutritional Immunity

Being an essential metal for humans, Mn is also essential for bacteria. Specifically, Mn is known to play a key role in the regulation of the general metabolism in *S. pneumoniae*, thus maintaining bacterial virulence [87]. Mn^2+^ cations are also required for Enterococcus faecalis virulence [88]. It has also been demonstrated that Mn^2+^ acquisition in *S. typhimurium* increases bacterial resistance to calprotectin and ROS-dependent killing, which is a factor in gut colonization [89]. Mn^2+^ is also involved in biofilm formation in *Bacilus subtilis* colonies [89].

The essentiality of Mn for both bacteria and host underlies the competence of the metal and its role in nutritional immunity. Mn is sequestered by the metal-binding neutrophil protein calprotectin through interaction with the His6 site, thus limiting its bioavailability for pathogenic bacteria [90]. In turn, bacteria have evolved Mn acquisition systems in order to cover the increasing requirement of metals [91].

Hypothetically, dysregulation of Mn handling and Mn overexposure may disrupt mechanisms of nutritional immunity, thus promoting infectious diseases as observed with iron overload [92]. Specifically, the disturbance of nutritional immunity with subsequent activation of bacterial pathogens may affect brain functioning through overproduction of specific bacterial toxins, altering neurotransmission and neuroactive metabolites, affecting both the peripheral and central nervous systems [93]. In addition, chronic bacterial infections are considered a risk factor for neurodegeneration [94].

## 7. Perspectives and Conclusions

**Figure 1:**
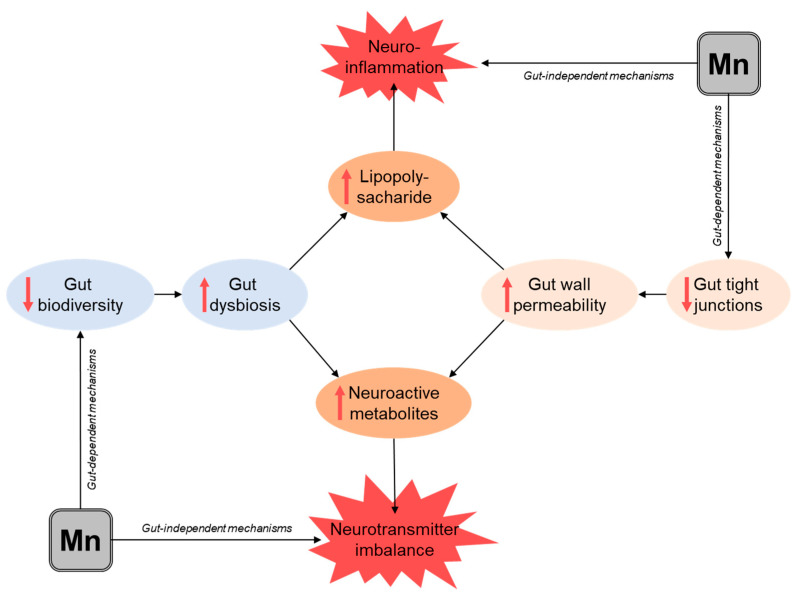
The proposed mechanisms linking Mn-induced alteration of gut integrity and intestinal microflora to Mn neurotoxicity. Mn overexposure was shown to affect relative abundance and biodiversity of gut microflora, ultimately resulting in impaired intestinal metabolomics. Mn-induced increase in Bacteroidetes abundance and a reduced Firmicutes/Bacteroidetes ratio may increase lipopolysaccharide levels. In turn, high doses of Mn may cause enterocyte toxicity, as well as affect gut wall integrity through disruption of tight junctions. The resulting increase in gut wall permeability further promotes increased translocation of LPS and neuroactive bacterial metabolites to the systemic blood flow ultimately leading to the brain, causing neuroinflammation and neurotransmitter imbalance. These gut-dependent effects may be potentiated by the gut-independent effects of Mn neurotoxicity.

Despite being insufficient, the existing data demonstrate the potential impact of Mn exposure on gut microbiota biodiversity. Given the role of bacterial metabolites in brain functions, Mn-induced perturbations in gut microflora may result in altered patterns of neuroactive metabolite production. In addition, increased levels of Gram-negative bacteria may result in systemic levels of LPS (endotoxin), which are known to induce inflammatory responses and possess neurotoxic efficacy. It is also notable that gut permeability plays a key role in the regulation of microbial metabolite systemic levels, and the impact of Mn overload on gut integrity may increase the entry of bioactive bacterial metabolites into systemic circulation and subsequently to the brain (Figure 1). This leads to the hypothesis that gut microbiota may be considered as a potential target for Mn toxicity.

In view of the increased risk of dietary Mn exposure, gut microbiota may be considered one of the early targets within the gastrointestinal tract that may mediate systemic effects of Mn exposure, including neurotoxicity, even prior to metal absorption. In turn, airborne Mn exposure is expected to affect gut microbiota to a much lower extent, and the contribution of gut dysbiosis to neurotoxicity may be significantly lower. Therefore, modulation of gut microbiota through therapeutic or preventive use of probiotics may be considered as one of the tools for the management of manganese toxicity in addition to earlier therapeutic approaches [1], as proposed for other toxic metals [95].

Although the existing data allow one to hypothesize the involvement of Mn-induced gut dysbiosis in Mn neurotoxicity, open questions include: (1) the estimation of the particular taxa of intestinal bacteria affected by Mn exposure; (2) the resulting alterations of bacterial metabolites translocated into circulation and their ability to modulate the gut–brain axis; and (3) the contribution of Mn-induced dysbiosis and bacterial lipopolysaccharide translocation to neuroinflammation. In addition, further studies should address the search for specific probiotic strains that might ameliorate Mn-induced perturbations in gut microflora and bacterial metabolite levels, and possibly reduce dietary Mn absorption.

At the moment, more detailed studies are required to fully characterize the interplay between Mn exposure and the gut, as well as its role in pathogenesis of neurodegeneration and other diseases.

## Data Availability

Not applicable.
